# Death of Prof. Briggs

**Published:** 1894-07

**Authors:** 


DEATH OF PROF. BRIGGS.—The faculty of the Medical Department of the University of Nashville and Vanderbilt University held a meeting on the 13th of June (ult.), when the President announced the death of Prof. W. T. Briggs. A committee was appointed and drafted resolutions expressive of the sentiments of the faculty touching the sad event, and testifying their high admiration for and appreciation of Prof. Briggs' many excellent qualities. He had been connected with that institution forty-three years, filling with ability and distinguished credit severally the positions of Demonstrator of Anatomy, Adjunct Professor Anatomy, Professor Surgical Anatomy and Physiology, Professor Obstetrics, and, for the last twenty-six years, the Chair of Principles and Practice of Surgery.

Not often, in the career of one man, has so varied and extensive a field of professional labors and duties been successfully cultivated amid the engrossing cares and exactions of a large and laborious private practice. In addition to all this he found time to make numerous and valued contributions to medical and surgical literature, and to deliver frequent addresses before scientific bodies.

His labors brought to him just and great ronown, and the profession he ornamented loved to honor him. He held the distinguished positions of President of his State Medical Association, President of the American Surgical Association, President of the Surgical Section Ninth International Medical Congress, and was a few years ago President of the American Medical Association. Surely the measure of his ambition must have been full; and a life of great beauty and usefulness was rounded off by the bestowal upon him of every honor which an admiring constituency could bestow; his death was like the plucking of ripened fruit. The medical profession of America mourn the loss of one of its most brilliant members.

